# A global perspective on the influence of environmental exposures on the nervous system

**DOI:** 10.1038/nature16034

**Published:** 2015-11-19

**Authors:** Desire Tshala-Katumbay, Jean-Claude Mwanza, Diane S. Rohlman, Gladys Maestre, Reinaldo B. Oriá

**Affiliations:** 1 Department of Neurology, Oregon Health & Science University, Portland, Oregon, 97239, USA; 2 National Institute of Biomedical Research, 1197 Kinshasa I, Democratic Republic of Congo; 3 Department of Neurology, University of Kinshasa, 825 Kinshasa XI, Democratic Republic of Congo; 4 Department of Ophthalmology, University of North Carolina at Chapel Hill, North Carolina 27599, USA; 5 Occupational and Environmental Health, The University of Iowa, Iowa 52242, USA; 6 Oregon Institute of Occupational Health Sciences, Oregon Health and Science University, Portland, Oregon, 97239, USA; 7 G.H. Sergievsky Center, Columbia University Medical Center, New York, New York 10032, USA; 8 Department of Morphology and Institute of Biomedicine, Faculty of Medicine, Federal University of Ceara, Fortaleza 60020, Brazil

## Abstract

Economic and social transitions in the era of globalization warrant a fresh look at the neurological risks associated with environmental change. These are driven by industrial expansion, transfer and mobility of goods, climate change and population growth. In these contexts, risk of both infectious and non-infectious diseases are shared across geographical boundaries. In low- and middle-income countries, the risk of environmentally mediated brain disease is augmented several-fold by lack of infrastructure, poor health and safety regulations, and limited measures for environmental protection. Neurological disorders may occur as a result of direct exposure to chemical and/or non-chemical stressors such as ultrafine particulate matters. Individual susceptibilities to exposure-related diseases are modified by genetic, epigenetic and metagenomic factors. The existence of several uniquely exposed populations, including those in the areas surrounding the Niger Delta or north western Amazon oil operations; those working in poorly regulated environments, such as artisanal mining industries; or those, mostly in sub-Saharan Africa, relying on cassava as a staple food, offers invaluable opportunities to advance the current understanding of brain responses to environmental challenges. Increased awareness of the brain disorders that are prevalent in low- and middle-income countries and investments in capacity for further environmental health-related research are positive steps towards improving human health.

Reports from the World Health Organization (WHO) indicate that the global burden of disease is determined by patterns of disease and disability in low- and middle-income countries (LMICs), which, predictably, have their own environmental signatures (http://www.who.int/healthinfo/global_burden_disease/about/en/). However, the impact of such signatures on both brain health and region or global disability-adjusted life years (DALYs) remains unknown and needs to be added to the agenda of global environmental health research. As for high-income countries, environmental health research programmes in LMICs must primarily focus on elucidating the entire range and source of exposures to define the human ‘exposome’ (the measure of all the exposures of an individual in their lifetime and how these exposures relate to health) in LMICs. The research agenda should include mechanistic and translational research, as well as capacity building to foster a new generation of environmental health scientists.

## SCOPE

In this Review, we focus on environmental risk factors for brain diseases and conditions in LMICs (http://data.worldbank.org/about/country-and-lending-groups). An iterative search of the literature was conducted using PubMed to retrieve information related to environmental determinants and mechanisms of brain disease in LMICs. Additional opinion was obtained from interviews with leading environmental scientists and neuroscientists, as well as programme officers at the US National Institutes of Health and US National Institute of Environmental Health Sciences (NIEHS), and Fogarty International Center. This Review integrates the goals and approaches to environmental health research as per the NIEHS 2012–2017 strategic plan (https://www.niehs.nih.gov/about/strategicplan/).

## ENVIRONMENTAL EXPOSURE AND BRAIN HEALTH

**Figure 1:**
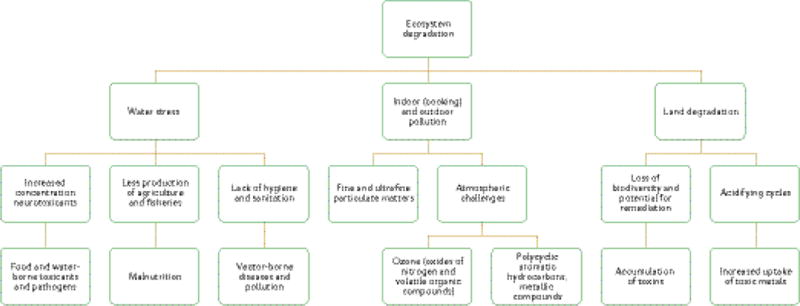
Environmental (chemical and non-chemical) threats to brain health in low- and middle-income countries. Multiple sources of exposure (air, water and food) coexist, and malnutrition and vector-borne diseases, notably infections, compound the risk of brain disease. Co-exposures not shown include heat, psychological stress and a poor physical environment, such as crowding.

LMICs are home to around 80–85% of the world’s population^1^. Of these 5.8 billion people^2^, 1 billion remain in extreme poverty, living below the US$1.25 per day poverty line^3^. Around 3 billion people do not have piped drinking water in their home and 173 million people rely on the direct use of surface water. Without proper sanitation, about one billion continue to defecate in gutters, in the open bush or in open water bodies^4^. Wildfires and deforestation are commonplace and drought and floods, possibly due to climate change, degrade the existing farming systems and create food insecurity^5–7^. Armed conflicts and population displacements impose an unnecessary toll on human life^8^. Industrial expansion coexists with an unprecedented rise in artisanal mining and unprotected labour^9^. In some instances, normal urbanization operations, such as road construction and quarantines (for example during Ebola outbreaks in the Democratic Republic of the Congo) have created conditions that exacerbated the risk of environmental exposure and brain disease^10^. Flawed regulations compounded by a lack of infrastructure set the stage for environmental degradation and pollution to pose serious threats — of a chemical or non-chemical nature — to human health. The degradation of local ecosystems leads to the creation of ‘microenvironments’ that have a high risk of harmful exposures, often resulting in unique challenges and increased risk of human disease (Fig. 1).

## HIGH-RISK POPULATIONS AND MICROENVIRONMENTS

Risk of exposure-related brain disease is determined by age, gender and microenvironments created by natural disasters in which economic, social and cultural determinants of health often have important roles. One example of a profit-mediated environmental risk is that caused by the oil industry through accidental spills or mismanagement of oil operations. For instance, crude oil operations have polluted large areas of rainforests, including streams and rivers in Ecuador, Peru and Colombia^11^. The population of Nigeria has faced similar challenges owing to reoccurring oil spills as a result of ageing, ill-maintained or sabotaged pipelines in the Niger Delta. The impact of such man-made and preventable natural disasters on human health has yet to be determined. Effects on human health will depend on the type and composition of the spilled oils, which often contain a mixture of polycyclic hydrocarbons that are known to be toxic to the nervous system^11^. Oil spills arise owing to reasons, such as a lack of vigilance, neglect of necessary health and safety checks, or sometimes even promotion of commercial interests at the expense of communities. Symptoms of acute exposure to raw oil include consistent episodes of headache, nausea, dizziness and fatigue. Chronic effects include psychological disorders, endocrine abnormalities and genotoxic effects^12^.

Microenvironments in which the population has a higher susceptibility to exposure-related diseases have also been created by extreme poverty and natural disasters, including drought and flooding that can degrade soils, plants and farming operations. The burden of conventional neurodevelopmental stressors (for example, lead) on children is exacerbated by unique environmental challenges, including malnutrition and enteric infections^13–16^ and, possibly, a diet of neurotoxicant-containing plants such as cassava (*Manihot esculenta*; also known as tapioca), the grass pea *Lathyrus sativus* or the seeds from the cycad plants, which are all known to be associated with a high-burden of neurodisabilities at a population level^17–22^. Populations with unique exposures and risks include those living in the tropical cassava belt of Angola, the Central African Republic, Cameroon, Democratic Republic of the Congo, Tanzania, Uganda, Nigeria and Mozambique^23–30^; those reliant on *L. sativus* as a staple food in Ethiopia, Eritrea, India and Bangladesh^20,31–33^; and the people of the Pacific island Guam or the Japanese Kii Peninsula where the rates of environmentally linked syndromes such as amyotrophic lateral sclerosis-parkinsonism-dementia complex (ALS/PDC) have been declining for reasons that have yet to be uncovered^34,35^.

The impact of early childhood diseases that lead to a vicious cycle of enteric infections and malnutrition has been underestimated and neglected, especially in areas that lack acceptable levels of hygiene and sanitation and that have reduced accessibility to vaccines and antimicrobials. This has caused clinically silent, chronic-illness-related effects, which jeopardize the child’s full cognitive development^13,15^. This vicious cycle establishes what is called environmental enteropathy, a mostly subclinical condition (even without diarrhoea) caused by various degrees of intestinal barrier dysfunction, luminal-to-blood intestinal bacterial translocation, low-grade local and systemic inflammation, and poor innate intestinal immune responses that may affect growth^36^ and cognition^37^ and possibly lead to neurodegeneration as well as liver, and metabolic diseases later in life^38,39^.

Adolescents in LMICs experience a higher burden of exposures (in contrast with those in high-income countries), primarily because of the childhood labour crisis. Although there are regulations and international agreements restricting child labour, often there are exceptions for certain industries, notably the growing agricultural industry, one of the most hazardous industries worldwide^40,41^. In this context, adolescent workers are at risk of exposure to agrochemicals such as pesticides^42,43^. Other work-related threats include exposure to organic solvents in work that involves painting and manufacture, to toxic metals and fine particulate matters in artisanal mining, and to heat and ambient air pollution while working long hours and outside^41^. Exposure to industrial solvents such as *n*-hexane, for example, may occur because of poor safety regulations or recreational glue sniffing. This may result in headache, acute encephalopathy or sensorimotor neuropathies that are reversible on cessation^44^.

Adults in LMICs may be at a particularly high risk of environmental exposure and related brain diseases compared with those in high-income countries. In general, adults experience a higher burden of disease owing to a lifetime of cumulative exposures and co-morbidities that are highly prevalent in LMICs. The latter include malaria, nutritional deficiencies and neurotropic infections such as those caused by human T-cell lymphotropic virus (HTLV). For example, it was reported that endemic foci of HTLV-I-associated myelopathy coexist with outbreaks of konzo, which causes spastic paraparesis and is linked to the toxicity of cassava cyanogens within some areas of the Democratic Republic of the Congo^45,46^. In certain circumstances, women of childbearing age are particularly vulnerable because they are more susceptible to the toxicity of cassava cyanogens for reasons that have not been elucidated, although may be linked to hormonal influences and poor nutrition^47^.

## PATHWAYS TO BRAIN DISEASE

Exposure-related brain damage may result from chemical and/or non-chemical stressors. Damage to the nervous system often leads to a range of bilateral and symmetrical motor and/or sensory symptoms. Behavioural problems, cognition deficits and psychiatric illness may also occur. Non-chemical stressors include, but are not limited to, psychological stress, heat, noise, fine and ultrafine particulate matter (FUPM), and waterborne, airborne or foodborne pathogens that may occur under the conceptual framework shown in Fig. 1. Chemicals with neurotoxic potential that people are commonly exposed to are listed in Tables 1–3. Mixed exposure, for example chemical-covered FUPM from industrial emissions; co-exposure to chemical and non-chemical stressors; and repeated and multiple exposure can occur, creating a complex human environmental exposome.

Brain damage linked to chemical exposure may result from chemicals interfering with neurotransmission through molecular mimicry or reacting with crucial biomolecules and causing incorrect function (for example, protein or DNA adduction and/or crosslinking). For both chemical and non-chemical exposures, the mechanisms of brain damage may include injury to the vascular system (for example, fine particulate-matter-induced vascular pathology), systemic dyshomeostasis (for example, cadmium-induced kidney disease) and hormonal imbalance (for example, through endocrine disruption; Table 1).

**Figure 2:**
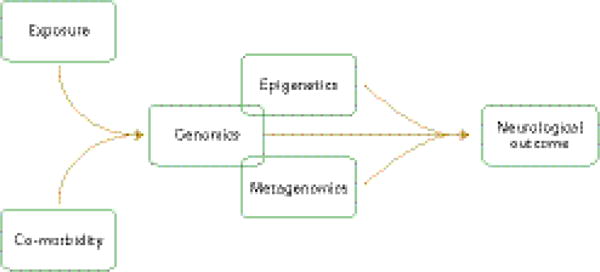
Environmental framework and pathways to environmentally induced neurological disease in low- and middle-income countries. Susceptibility to neurological disease is determined at the interface between a particular exposure, epigenetic and metagenetic make up, and the presence of co-morbidities.

The susceptibility to exposure-related disease is, however, determined by mechanisms of functional genetics, epigenetics and metagenomics at the interface between risk factors and neurological outcomes (Fig. 2).

It is increasingly acknowledged that genetic and epigenetic factors, including the effect of maternal stress on brain function, influence the effect of environmental exposure^48,49^. For example, the E4 allele of the *APOE* gene that is reportedly associated with higher risk of late-onset Alzheimer’s disease, although not in people from sub-Saharan Africa and with a mild association among Hispanic people, is also associated with protection against early childhood diarrhoea and its related cognitive impairment^50–52^. One example of gene–environment interactions is the relationship between air pollution components and the gene encoding the MET receptor tyrosine kinase. Several studies have implicated *MET* as an autism risk gene^53–55^. Stratification of the risk conferred by a functional promoter variant in this gene (rs1858830) and by local traffic-related air pollution (regional particulate matter less than 10 microns in diameter and nitrogen dioxide exposure) revealed significant multiplicative interaction between the risk genotype and the air pollution exposure^56^.

Our knowledge of the pathways that lead to late onset of exposure-related neurological disease is still sparse^57,58^. Many studies suggest that the genetic and environmental causes of late onset diseases act in parallel and share common molecular mechanisms^59^. A number of studies have supported the concept that early-life exposure to pollutants reprograms global gene expression in old age through epigenetic mechanisms^60–63^. Variation in exposure response, even among individuals exposed to the same environment could be due not only to early-life exposures, but also to differences in genetic make up^64–66^. The extent and nature of exposures and related brain diseases in LMICs provide opportunities to explore and overcome the long reach that childhood exposure has into adulthood, as well as provide us with new advances in environmental health sciences^67^.

**Figure 3:**
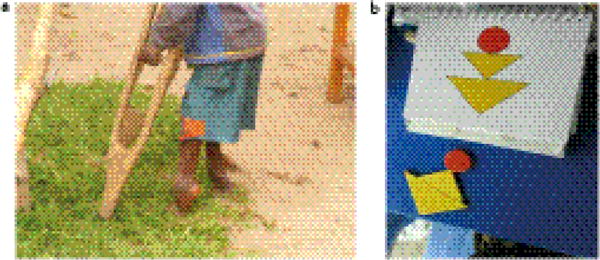
Neurocognition deficits in konzo, a disease linked to eating cyanogenic cassava. **a**, Spasticity in a 14-year old boy severely affected by konzo. **b**, Deficits in mental processing are evident from the results of a neuropsychological test.

Exposure-related neurological deficits in LMICs range from peripheral neuropathies to a large number of acute, subacute or chronic central nervous system diseases. Deficits may occur prenatally, or during childhood or adolescence, and may be carried through to old age. Clinical implications include, but are not limited to, neural tube defects, learning disabilities, behavioural problems, psychiatric disorders, cognitive decline and the occurrence of distinct entities such as neurolathyrism, tropical ataxic neuropathy, ALS/PDC and konzo^20,30,35,68,69^ (Fig. 3).

The human microbiome may be of particular interest to the mechanistic understanding of exposure-related diseases in LMICs because it may influence the burden of heavy metals^70^, the metabolism of food-borne neurotoxicants such as cassava cyanogens^18^, and the outcome of enteric diseases in early life, including the child’s neurodevelopmental potential^71–73^.

## RESEARCH AND CAPACITY BUILDING

Recent advances in environmental health sciences have elucidated the myriad risk factors and mechanisms of brain damage that are associated with environmental exposures. The existence of uniquely exposed populations in LMICs offers invaluable opportunities to advance our current understanding of brain responses to environmental threats. In some instances, well-characterized neurotoxicants may be used as chemical probes to dissect the pathophysiology of the nervous system. Challenges at the population level still remain, including setting exposure limits and developing metrics and methodologies to assess the long-term impact of environmental exposures on disease burden in LMICs and, therefore, globally. Climate change and mining of rare elements, which may include radioactive materials, present unpredictable risks, and should be added to the environmental health research agenda. The toll of such exposures on the global burden of disease may be efficiently addressed only through competent partnerships and alliances established on a global scale and focused on key areas and priorities (Box 1). Although there is evidence that some of these are already in place, more research and research capacity is needed to continue this agenda to improve human health, globally.

## ONE HEALTH–GLOBAL HEALTH DIMENSIONS

Environmental degradation and contamination, changes in climate and ecosystems, and vector-born pathogens or neurotoxicants are the primary environmental threats to human life and intellectual performance. Humans, plants and animals adapt to environmental challenges, but some may overcome their adaptive capabilities and create imminent risks for all^17,74^. Addressing environmental threats and risks for incapacitating diseases will therefore require a serious commitment to trans-disciplinary work, vigilance and capacity building on a global scale^75^

## Figures and Tables

**Table 1:** Heavy metals and exposure-related outcomes

Heavy metal	Source of exposure	Susceptibility window	Neurological outcomes	Proposed mechanisms
**Lead**^76–78^	Lead-contaminated dust, lead-based paint, soil, drinking water, air, leaded gasoline, toys and lead-contaminated sweets	Lifelong	Visual and verbal memory decline, intellectual deficits, decline in executive functioning (fine motor function, hand-eye coordination and reaction time) and hyperactivity in children	Disruption of neurotransmitter release and function, and prenatal disruption of neuronal migration and differentiation. Aggravating factors include poor nutrition (deficiency in iron, zinc and calcium) and younger age.
**Mercury**^79,80^	Mining industry, power plants, crematoria, charcoal industry, and contaminated food (mostly sea food) and water	From neural development to neurulation, and adolescence	Ataxia in adults and language, attention, and visuospatial performance deficits in children	Oxidative stress or impairment of intracellular calcium and glutamate homeostasis
**Arsenic**^80,81^	Contaminated food and drinking water, air and arsenic-based treatments	5–15 years	Impaired selective and focused attention and long-term memory in children, and sensorimotor polyneuropathy	Oxidative stress or disruption of metabolism of neurotransmitters
**Copper**^82^	Contaminated drinking water and food, uncoated copper cookware and infant formula containing copper	Children Those over 65	Alzheimer’s disease, OCD, ADHD, antisocial behaviour and anxiety in children	Oxidative stress, microglia cell activation or promotion of α-synuclein and fibril formation
**Cobalt**^82,83^	Contaminated drinking water and food, inhalation of dust containing cobalt particles in various industries	Prenatal, young children and the elderly	Optic, auditory and peripheral neuropathy, motor deficits and verbal memory loss	Alteration of mitochondrial oxidative phosphorylation or depletion of neurotransmitters
**Cadmium**^83^	Fumes or dust, cigarette smoke, and contaminated food and water	Prenatal, young children and the elderly	Antisocial behaviour and attention impairment in children, parkinsonism and peripheral neuropathy	Oxidative damage and neurotransmitter disruption
**Manganese**^76,79^	Airborne as fumes, aerosols or suspended particulate matter and contaminated water	Childhood and the elderly	Reduced IQ, impaired verbal learning and working and immediate memory in children, and Parkinson-like symptoms	Disruption of mitochondrial respiratory chain reaction. Aggravating factors include iron deficiency and impaired biliary excretion (liver injury or disease).
**Aluminium**^84^	Contaminated air, water and food, cosmetics (such as antiperspirants), metal industries and pharmaceuticals	Lifelong	Alzheimer’s pathology in the form of neurofibrillary tangles	Disruption of mitochondrial respiratory chain reaction or inflammation. Zinc deficiency acknowledged as an aggravating factor.

ADHD, attention-deficit hyperactivity disorder; OCD, obsessive compulsive disorder.

**Table 2:** Organic compounds and exposure-related outcomes

Organic compound	Source of exposure	Susceptibility window	Neurological outcomes	Proposed mechanisms
**Bisphenol A**^85,86^	Food from cans with linings that contain BPA, and contaminated food and water	Prenatal and childhood	Anxious behaviour, hyperactivity and depressive behaviour, and learning impairment in children	Unclear but females seem to be more susceptible
**Phthalates**^86–88^	Food or drink that has been in contact with containers or products containing phthalates, and air and dust containing phthalates	Prenatal and childhood	Depressive and conduct-related behaviours (ODD, attention problems, rule-breaking and aggressive behaviour in children)	Oxidative stress
**Organophosphates**^89,90^	Contaminated food and water, polluted air and professional dermal contact	Lifelong	Neurodevelopmental deficits, impaired attention and working memory, impaired speed and executive functions, and delayed peripheral polyneuropathy	Inhibition of acetyl-cholinesterase
**Organochlorinated compounds (DDT/PCBs)**^91^	Contaminated food, drinking water and air	Prenatal and lifelong	Impaired intellectual ability, ADHD- like behaviours and locomotor deficits	Disruption of neurotransmitter function, oxidative stress or derangement of calcium homeostasis. Children seem to be more susceptible.
**Organobromide compounds (PBDEs)**^92,93^	Contaminated food, water and air	Lifelong	IQ deficits, impaired attention, fine motor coordination and cognition functioning in children	Impairment of thyroid hormone homeostasis
**Organic solvents**^94,95^	Air and professional dermal contact and glue sniffing	Adolescents and adults	Headache, memory deficits, and central and peripheral neuropathy	Protein adduction or oxidative stress misfolding
**Food-born neurotoxicants (cassava cyanogenic glucosides and BOAA in** *Lathyrus sativus*)^96–98^ **or contaminants (fungal toxins)**	Oral ingestion	Lifelong	Spastic paraparesis, cognition deficits and epilepsy	Oxidative stress, excitotoxicity and protein carbamylation for cassava cyanogens. Children and females seem to be more susceptible. Malnutrition is acknowledged as an aggravating factor

ADHD, attention-deficit hyperactivity disorder; BOAA, beta-(N)-oxalyl-amino-L-alanine acid; BPA, bisphenol A; DDT/PCBs, dichlorodiphenyltrichloroethane/polychlorinated biphenols; ODD, oppositional defiant disorder.

**Table 3:** Complex exposures and neurological outcomes

Exposure	Source of exposure	Susceptibility window	Neurological outcomes	Proposed mechanisms
**Coal/charcoal burning**^99,100^	Charcoal/coal combustion, gas grilling, wood smoke, or coal mine dust or ash	Lifelong	Neurological signs of exposure to arsenic	Oxidative stress
**Car emissions**^101,102^	Contaminated air	Lifelong	Learning disability and motor impairment	Oxidative stress or neurotransmitter disruption
**Fine and ultrafine particulate matters**^103^	Air pollution from car or construction equipment exhausts, wood burning, heating oil or coal, forest fires, volcanic eruptions, tobacco smoke and cooking	Lifelong	Behavioural and decreased IQ, impaired fluid cognition, memory and executive functions, and possibly autism	Oxidative stress, neurotransmission disruption or neuroinflammation

## BOX 1 INTERWOVEN RESEARCH AREAS AND INVESTMENT PRIORITIES IN GLOBAL ENVIRONMENTAL RESEARCH

- Epidemiology and statistical modeling for exposure and risk assessment in co-exposure and co-morbidity scenarios
- High-throughput ‘-omic’ methodologies
- Bioinformatics and knowledge management
- Development of diagnostic and remediation tools — validation and implementation of environmental sensors, detectors and biomonitors of exposure and related outcomes
- Development of metrics and methodologies to assess the long-term impact of environmental exposures on neurological disease burden
- Understanding the pathways that lead to late onset of exposure-related neurological diseases
- Training and capacity building in the areas listed above
